# Robert Arthur Askins

**Published:** 1935

**Authors:** 


### Obituary

---

ROBERT ARTHUR ASKINS, Barrister-at-Law (Gray's Inn), M.A., M.D. (T.C.D.), D.P.H. (Camb.).

The tragic death of Dr. R. A. Askins, principal medical officer to the Government of Southern Rhodesia, whilst returning to South Africa, has deprived two continents of a well-known and distinguished figure in public health. His untimely death is particularly mourned in Bristol, where he had many friends, and had lived and worked, first as School Medical Officer, and later as Medical Officer of Health of the city, port and school service.

Dr. Askins was born in Clonmore, County Carlcw, Ireland, in 1880. He received his medical education at Trinity College, Dublin, and graduated M.B., Ch.B., B.A.O., at Dublin University in 1907 (First Class Honourman and First Place Final Medical Examination). Later he gained the Cambridge Diploma in Public Health and the degrees of M.A. and M.D. at Dublin University. He was also a barrister-at-law (Gray's Inn).

After spending some time in gaining clinical experience he turned to public health, and in 1908 was appointed Assistant Medical Officer of Health for Merthyr Tydfil. From 1909 to 1914 he served as Assistant Medical Officer to Lancashire Education Committee. In 1914 he came to Bristol as the first full-time School Medical Officer under the Bristol Education Committee. He soon showed himself to be an able administrator and a capable leader, and the development of the School Medical Service in Bristol is a lasting tribute to his organising ability.

During the Great War he served with the British Expeditionary Force (1915-19) as Specialist Sanitary Officer with the rank of Captain, R.A.M.C. (T.), and was mentioned in despatches. He returned to his former post as School Medical Officer at the termination of the War, and in 1924 was appointed Deputy Medical Officer of Health for the city. On the retirement of Dr. D. S. Davies in 1928 he was promoted to be Medical Officer of Health, in addition to retaining his position as School Medical Officer, and for the first time the two services became co-ordinated under one head. He was also appointed Lecturer in Public Health at the University.

Great changes were occurring in local health government during these two years, and Dr. Askins was actively engaged in preparing the way for the re-organisation in accordance with the Local Government Act, when in May, 1930, he resigned his post upon his appointment as principal medical officer to the Government of Southern Rhodesia.

In public life Dr. Askins was much respected for his ability and his determination. To his friends he was known to be of a shy and retiring disposition. He was extremely fond of simple rural life, and whenever his duties permitted he sought the company of the Somerset and Gloucester hills.

He leaves a widow and two young children, to whom our deepest sympathy is extended.

